# Cytotoxic Natural Products from Marine Sponge-Derived Microorganisms

**DOI:** 10.3390/md15030068

**Published:** 2017-03-10

**Authors:** Huawei Zhang, Ziping Zhao, Hong Wang

**Affiliations:** School of Pharmaceutical Sciences, Zhejiang University of Technology, Hangzhou 310014, China; baixl2012@163.com (Z.Z.); yingzhoudengyuan@sina.com (H.W.)

**Keywords:** marine microbe, sponge-derived microbe, natural product, cytotoxic compound

## Abstract

A growing body of evidence indicates that marine sponge-derived microbes possess the potential ability to make prolific natural products with therapeutic effects. This review for the first time provides a comprehensive overview of new cytotoxic agents from these marine microbes over the last 62 years from 1955 to 2016, which are assorted into seven types: terpenes, alkaloids, peptides, aromatics, lactones, steroids, and miscellaneous compounds.

## 1. Introduction

The search for cytotoxic agents from marine resources has always attracted the attention of natural products chemists [1,2]. More than 10% of the screened marine sponges display cytotoxic activities [3,4,5]. Marine sponges are well known to be hosts for a large community of microorganisms, which comprise a significant percentage (up to 50%–60%) of the biomass of the sponge host [6,7]. A growing body of evidence has indicated that marine sponges undergo symbiotic relationships with microbes such as bacteria and fungi, which are likely to be the prolific producers of bioactive secondary metabolites [8,9]. This review provides a comprehensive overview of 107 new cytotoxic agents metabolized by marine sponge-derived microbes, which are assorted into seven types, including terpenes, alkaloids, peptides, aromatics, lactones, steroids, and miscellaneous compounds discovered from 1955 to 2016.

## 2. Terpenes

### 2.1. Sesquiterpenes

**Chart 1:**
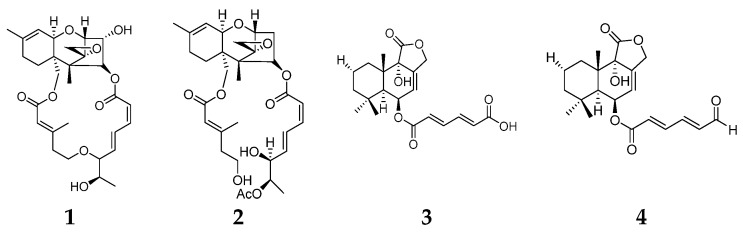
Chemical structures of compounds **1**–**4**.

**Chart 2:**
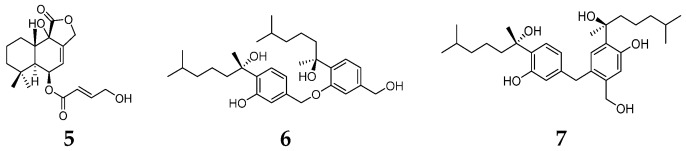
Chemical structures of compounds **5**–**7**.

Two new trichothecenes (Chart 1), 3-hydroxyroridin E (**1**) and 13′-acetyltrichoverrin B (**2**), were isolated from *Myrothecium verrucaria* associated with a *Spongia* sp. (Coll. No. 97103) collected from the coast of Maui, HI, USA, and were shown to have potent cytotoxicity against murine lymphocytic leukemia L1210 and human colon tumor H116 cell lines [10]. Chemical investigation of a marine-derived fungus *Aspergillus ustus* from the sponge *Suberites domuncula* (collected from the Adriatic Sea) led to isolation of two new drimane sesquiterpenoids **3** and **4** (Chart 1). Bioassay results indicated that these sesquiterpenes exhibited potent inhibitory effect on tumor cell lines L5178Y, HeLa, and PC12 with half maximal effective concentration (EC_50_) values ranging from 0.6 to 5.3 µg/mL [11].

(*E*)-6-(40-hydroxy-20-butenoyl)-strobilactone A (**5**), isolated from *Aspergillus insuetus* (OY-207) colonizing in a Mediterranean sponge *Psammocinia* sp., was shown to have a cytotoxic effect on the MOLT-4 cell line by 55% at 50 mg/mL [12]. Two new dimers of phenolic bisabolane sesquiterpenoid **6** and **7** (Chart 2) were metabolized by a marine-derived fungus *Aspergillus* sp. associated with the sponge *Xestospongia testudinaria*, which was collected around the South China Sea. Compound **6** exhibited in vitro moderate cytotoxicity against human hepatoma cell line HepG-2 and human cervical cell line Caski with half maximal inhibitory concentration (IC_50_) values of 9.31 and 12.40 µg/mL, while **7** showed selective activity with IC_50_ values of 2.91 and 10.20 µg/mL, respectively [13].

**Chart 3:**
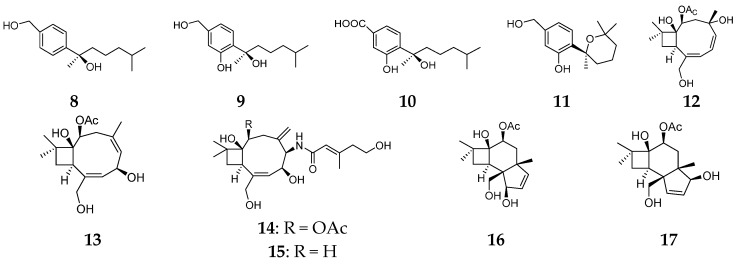
Chemical structures of compounds **8**–**17**.

Four new bisabolane-type sesquiterpenoids (**8**–**11**) were found in the fermentation broth of *Aspergillus* sp. in the marine sponge *Xestospongia testudinaria* from the South China Sea (Chart 3). Biological assay suggested that these compounds were weakly cytotoxic (IC_50_ value >50 µg/mL) against human promyelocytic leukemia HL-60 and human lung carcinoma A-549 [14]. The marine fungus *Hansfordia sinuosae* derived from the sponge *Niphates* sp., was shown to produce six new caryophyllene-based sesquiterpenoids, punctaporonins H–M (**12**–**17**) (Chart 3). However, these sesquiterpenoids possessed weak cytotoxicities against human colon carcinoma HCT-8, human hepatoma Bel7402, human gastric carcinoma BGC823, human lung adenocarcinoma A549, and human ovarian carcinoma A2780 with IC_50_ values >10 µM [15].

### 2.2. Sesterterpenoids

**Chart 4:**
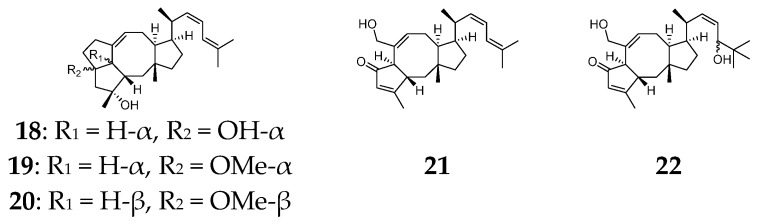
Chemical structures of compounds **18**–**22**.

Chemical examination of the marine fungus *Aspergillus ustus* isolated from a Mediterranean sponge *Suberites domuncula* yielded five new ophiobolin-type sesterterpenoids **18**–**22** [16] (Chart 4). These compounds were assayed for their cytotoxic activity against the murine lymphoma cell line L5178Y at 10 mg/mL.

### 2.3. Diterpenes

**Chart 5:**
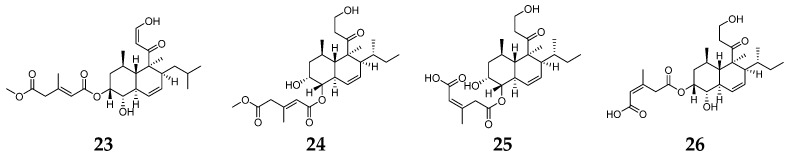
Chemical structures of compounds **23**–**26**.

Four novel decalin derivatives, tandyukisins (**23**–**26**), were produced by a strain of *Trichoderma harzianum* OUPS-111D-4 originally derived from the marine sponge *Halichondria okadai* collected in Osaka Bay, Japan (Chart 5). Cytotoxic assays suggest that compound **23** exhibited moderate cytotoxicity against murine leukemia cell lines P388 and L1210 and human leukemia cell line HL-60. Compounds **24**–**26** had moderate cytotoxicity against a disease-oriented panel of 39 human cancer cell lines (HCC panel). However, these diterpenes showed slightly selective growth inhibition against the central nervous system cancer SNB-75 cell line in the HCC panel [17,18].

### 2.4. Meroterpenoids

**Chart 6:**
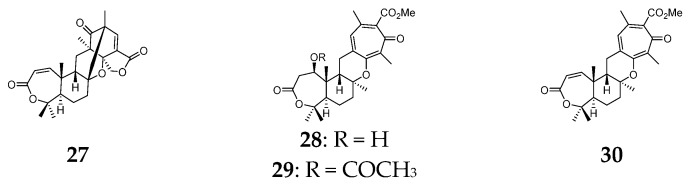
Chemical structures of compounds **27**–**30**.

Chemical investigation of the EtOAc extract of the culture medium of the marine-derived fungus *Aspergillus insuetus* OY-207 led to the isolation of a novel meroterpenoid, insuetolide C (**27**) (Chart 6). The strain OY-207 was isolated from a Mediterranean sponge *Psammocinia* sp. (collected approximately 200 m off-shore from Sdot-Yam, Israel). Compound **27** exhibited mild cytotoxicity towards human leukemia MOLT-4 cells [12]. Another *Aspergillus* strain derived from an unidentified sponge (collected at Manele Bay, HI, USA) was found to metabolize three new cytotoxic meroterpenoids, tropolactones A–C (**28**–**30**) (Chart 6), which showed weak cytotoxicity against human colon adenocarcinoma cells (HCT-116) with IC_50_ values of 13.2, 10.9 and 13.9 µM, respectively [19].

## 3. Alkaloids

**Chart 7:**
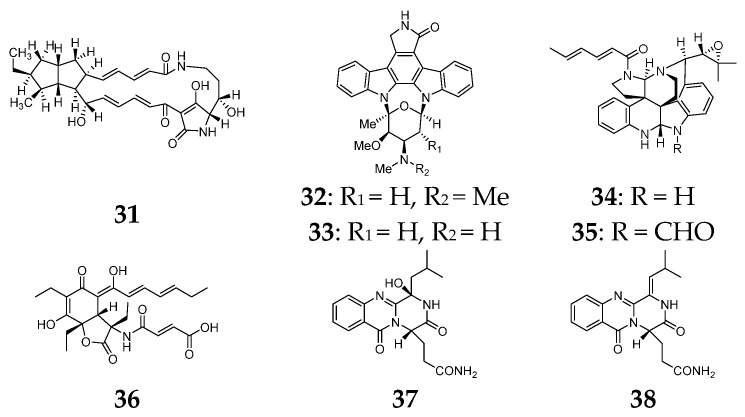
Chemical structures of compounds **31**–**38**.

Chemical examination of the cultured mycelium of a bacterium *Alteromonas* sp. from the sponge *Halichondria okadai* led to the isolation of one novel tetracyclic alkaloid: **31** (Chart 7). It exhibited cytotoxicity against the murine leukemia cell line P388, murine lymphoma L1210, and human epidermoid carcinoma KB cells in vitro with IC_50_ values of 0.1, 1.7, and 5.0 μg/mL, respectively [20]. 4′-*N*-methyl-5′-hydroxystaurosporine (**32**) and 5′-hydroxystaurosporine (**33**) (Chart 7) were obtained from a marine strain of *Micromonospora* sp. L-31-CLCO-002, a symbiont on the sponge *Clathrina coriacea*, and in vitro were shown to have strong cytotoxic activities against tumor cell lines P388D1 (ATCC CCL-46), A549 (ATCC CCL-185), HT-29 (ATCC HTB-38), and SK-MEL-28 (ATCC HTB-72) [21].

Two new congener alkaloids, communesins **34** and **35** (Chart 7), were detected in the ethyl acetate extract of a *Penicillium* sp. which was isolated from the Mediterranean sponge *Axinella verrucosa*. Communesin **34** was observed to be most active on the human acute T lymphoblastic leukemia cell line MOLT-3 with an ED_50_ value of 8.6 µg/mL. Conversely, **35** possessed a strong inhibitory effect on the human acute B lymphoblastic leukemia cell line SUP-B15 with an ED_50_ value of 9.0 µg/mL [22]. A new sorbicillin-derived compound, **36**, metabolized by *Penicillium chrysogenum* associated with the Mediterranean sponge *Ircinia fasciculata* was found to exhibit a strong cytotoxic activity against L5178y cells and low toxicity to cervical carcinoma HeLa S3 cells and pheochromocytoma PC12 cells [23]. Another *Penicillium* strain, *P.* *aurantiogriseum* SP0-19, was isolated from the marine sponge *Mycale plumose* and shown to produce two novel quinazoline alkaloids: aurantiomides **37** and **38**. Compound **37** exhibited moderate cytotoxic activities against tumor cell lines HL-60 and P388 with IC_50_ values of 52 and 54 μg/mL, respectively, while **38** selectively inhibited BEL-7402 and P388 cell lines with IC_50_ values of 62 and 48 μg/mL, respectively [24].

**Chart 8:**
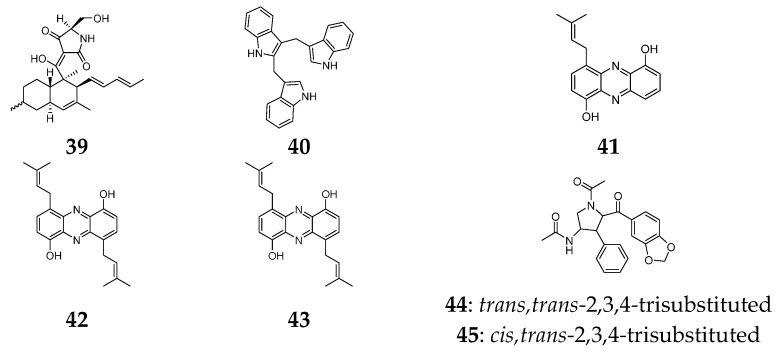
Chemical structures of compounds **39**–**45**.

Chemical study of the marine-derived fungus *Beauveria bassiana* from the North Sea sponge *Myxilla incrustans*, afforded a new equisetin-like tetramic acid derivative beauversetin (**39**) with moderate activity against an unknown tumor cell line [25] (Chart 8). A novel indole oligomer (**40**) (Chart 8) was metabolized by a *Psychrobacter* strain isolated from the marine sponge *Stelletta* sp. (collected from the coast of Geoje Island) and was shown to have an inhibitory effect on five human solid tumor cell lines: A-549, SK-OV-3, SK-MEL-2, XF-498, and HCT-15 with EC_50_ values of 2.34, 1.57, 3.44, 2.39, and 3.13 mg/mL, respectively [26]. Three novel alkaloids, JBIR-46 (**41**), -47 (**42**), and -48 (**43**) (Chart 8), were detected in the cultures of bacterium *Streptomyces setonensis* SpC080624SC-11 and SpA080624GE-02, which were isolated from the marine sponge *Cinachyra* sp. and *Stylotella aurantium* (collected from the sea near Tateyama, Chiba Prefecture). Cytotoxic tests indicated that compounds **41**–**43** possessed weakly cytotoxic activities against human acute myelogenous leukemia HL-60 cells with IC_50_ values of 189, 226, and 96 μM, respectively [27]. Chemical examination of a marine fungus *Aspergillus ustus*, isolated from the Mediterranean sponge *Suberites domuncula*, yielded two new pyrrolidine alkaloids, **44** and **45** (Chart 8), which showed weak cytotoxicty against murine lymphoma L5178Y cells at 10 mg/mL [16].

**Chart 9:**
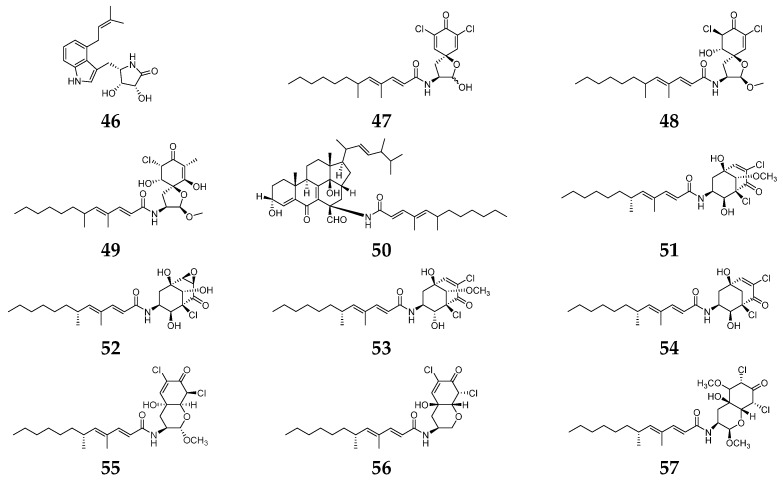
Chemical structures of compounds **46**–**57**.

One new secondary metabolite, amycolactam (**46**) (Chart 9), was isolated from a rare actinomycete *Amycolatopsis* sp. colonized in an unidentified sponge gathered from Micronesia. It had a broad spectrum of cytotoxic activities against SNU638 and HCT116 with IC_50_ values of 0.8 and 2.0 μM, respectively, and against A546, K562, and SK-HEP1 cells with IC_50_ values of 13.7, 9.6, and 8.3 μM, respectively [28]. Ten novel cytotoxic compounds (**47**–**56**) (Chart 9) were produced by a strain of *Gymnasella dankaliensis*, a symbiont on a *Homaxinella* marine sponge (collected in the Osaka Bay of Japan). All these natural products exhibited cytotoxic activities against the lymphocytic leukemia P388 cell line (ED_50_ 18.0, 10.8, 10.6, 10.1, 0.13, 0.03, 1.7, 2.8, 0.15, and 0.16 µg/mL, respectively). Furthermore, compound **53** had appreciable growth inhibition against tumor cell lines BSY-1 (breast) and MKN7 (stomach) lines (log GI_50_: −5.47 and −5.17, respectively) [29,30,31,32]. Continuous investigation of secondary metabolites produced by the *Homaxinella* derived fungus, *Gymnascella dankaliensis*, yielded a new compound dankastatin C (**57**) (Chart 9). This alkaloid exhibited an ED_50_ value of 57 ng/mL against the murine lymphocytic leukemia P388 cell line, which was as potent as that of 5-fluorouracil (ED_50_ 78 ng/mL) [33].

## 4. Peptides

**Chart 10:**
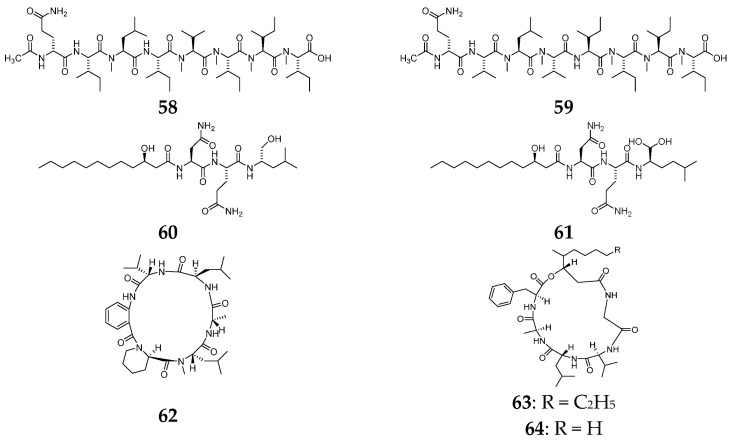
Chemical structures of compounds **58**–**64**.

Two highly *N*-methylated linear octapeptides, RHM1 (**58**) and RHM2 (**59**) (Chart 10), were produced by an atypical strain of *Acremonium* sp. cultured from a *Teichaxinella* sp. marine sponge (collected in Papua New Guinea) and were shown to have mild cytotoxicity against murine L1210 cells by a disk diffusion soft agar colony-forming assay [34]. The investigation of the chemical constituents of the mycelia and culture filtrate of a fungus *Aspergillus versicolor* from a marine sponge *Petrosia* sp. (collected off the coast of Jeju Island, Korea) yielded two novel lipopeptides: fellutamide C (**60**) and fellutamide F (**61**) (Chart 10). Compound **61** exhibited strong cytotoxiciy against human lung cancer A549, human ovarian cancer SK-OV-3, human skin cancer SK-MEL-2, human central nervous system (CNS) cancer XF498, and human colon cancer HCT15 [35,36]. One fungal strain, *Aspergillus similanensis* KUFA0013, derived from the sponge *Rhabdermia* sp. (Similan Islands, Thailand) was found to produce a new cyclohexapeptide, similanamide (**62**) (Chart 10), which possessed in vitro weak inhibitory activity against breast adenocarcinoma MCF-7, non-small cell lung cancer NCI-H460, and melanoma A373 cell lines [37]. Chemical examination of *Scopulariopsis brevicaulis* from the marine sponge *Tethya aurantium* (Limski Fjord, Croatia) afforded two novel cyclodepsipeptides: scopularides A (**63**) and B (**64**) (Chart 10). Bioassay tests suggested that compounds **63** and **64** significantly inhibited growth of three tumor cell lines. At a concentration of 10 µg/mL, the viability of the cell lines Colo357, Panc89 (pancreatic tumor cells), and HT29 (colon tumor cells) was reduced by 36% (**63**) and 26% (**64**), 42% (**63**) and 49% (**64**), and 37% (**63**) and 24% (**64**), respectively [38].

## 5. Aromatics

### 5.1. Polyketides

**Chart 11:**
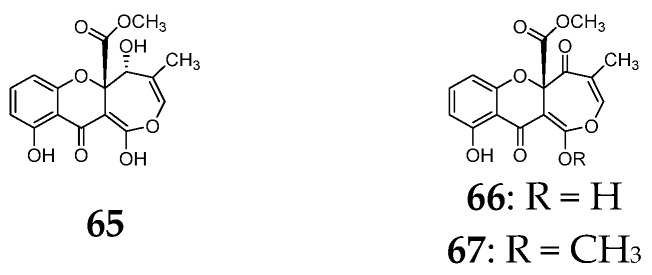
Chemical structures of compounds **65**–**67**.

Chemical investigation of *Penicillium brocae*, obtained from a tissue sample of a Fijian sponge *Zyzyya* sp., led to the isolation of three novel polyketides: brocaenols A–C (**65**–**67**) (Chart 11). Compounds **65**, **66** and **67** showed moderate antiproliferative effects on the HCT-116 cell line with IC_50_ values of 20, 50, and >50 µg/mL, respectively [39].

### 5.2. α-Pyrone Derivatives

**Chart 12:**
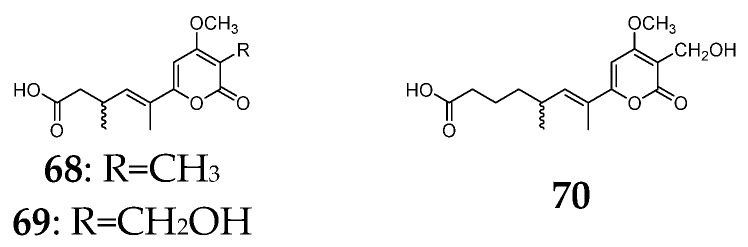
Chemical structures of compounds **68**–**70**.

Three new α-pyrone derivatives (**68**–**70**) (Chart 12), were characterized from *Petriella* sp. associated with one Mediterranean sponge, *Suberites domuncula*. Compound **68** exhibited pronounced cytotoxic activity against the L5178Y cell line, while congeners ***69*** and ***70*** had moderate activity [40].

### 5.3. Anthraquinones

**Chart 13:**
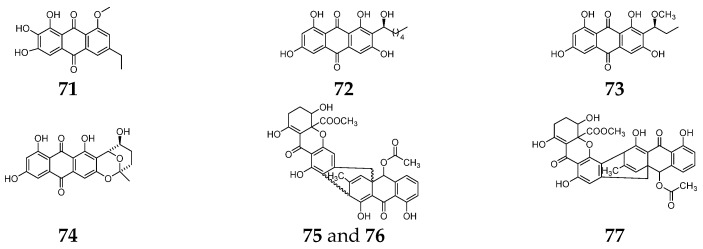
Chemical structures of compounds **71**–**77**.

From a strain of the fungus *Emericella variecolor* derived from the marine sponge *Haliclona valliculata* (collected at Secca di Capo di Fonza, Elba, Italy), a new natural product called evariquinone **71** (Chart 13) was isolated and found to display antiproliferative activity towards tumor cell lines KB (60% inhibition) and NCI-H460 (69% inhibition) at 3.16 mg/mL [41]. Study on the bioactive metabolites of *Aspergillus versicolor* derived from a marine sponge *Petrosia* sp. (Jeju Island, Korea) afforded three anthraquinones (**72**–**74**) (Chart 13) by bioactivity-guided fractionation. Those metabolites exhibited significant cytotoxicity against five human solid tumor cell lines (A-549, SK-OV-3, SK-MEL-2, XF-498, and HCT-15) with IC_50_ values in the range of 0.41–3.88 µg/mL [42]. Three new compounds, JBIR-97 (**75**), -98 (**76**), and -99 (**77**) (Chart 13) were produced by a fungal strain *Tritirachium* sp., SpB081112MEf2, derived from the sponge *Pseudoceratina purpure* (collected from offshore sites in Sakuraguchi, Ishigaki Island, Okinawa Prefecture, Japan). By the water-soluble tetrazolium-8 (WST-8) colorimetric assay, compounds **75**, **76**, and **77** were shown to have cytotoxic effects on HeLa cells (IC_50_: 11, 17, and 17 µM, respectively) and ACC-MESO-1 cells (IC_50_: 31, 63, and 59 µM, respectively) [43].

### 5.4. Bicoumarin

**Chart 14:**
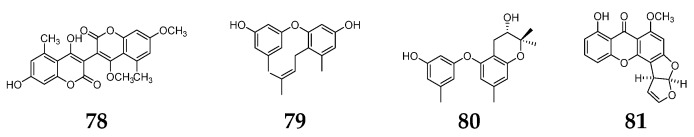
Chemical structures of compounds **78**–**81**.

Fractionation of the EtOAc extract of a static culture of *Aspergillus niger* from a Mediterranean sponge *Axinella damicornis,* yielded one new secondary metabolite: 3,3′-bicoumarin bicoumanigrin (**78**) (Chart 14). MTT assay indicated that this compound exhibited moderate inhibitory effects on the growth of leukemia and carcinoma cell lines using incorporation of ^3^H-thymidine as a marker [44].

### 5.5. Ethers

Two new prenylated diphenyl ethers (**79** and **80**) (Chart 14) were purified from the fungus strain of *Aspergillus versicolor* Hmp-F48 associated with marine sponge *Hymeniacidon perleve*. Compounds **79** and **80** showed moderate inhibitory activities against the human promyelocytic leukemia cell line HL-60 with IC_50_ values of 6.35 and 19.97 µM, respectively [45].

### 5.6. Xanthones

Chemical analysis of the fungal strain *Aspergillus versicolor* derived from a marine sponge *Petrosia* sp. (collected from the coast of Jeju Island, Korea), afforded a new xanthone **81** (Chart 14), which had strong cytotoxic activity against five human tumor cell lines (A-549, SK-OV-3,SK-MEL-2, XF-498, and HCT-15) with IC_50_ values ranging from 1.22 to 4.61 µg/mL [42].

### 5.7. Other Aromatic Compounds

**Chart 15:**
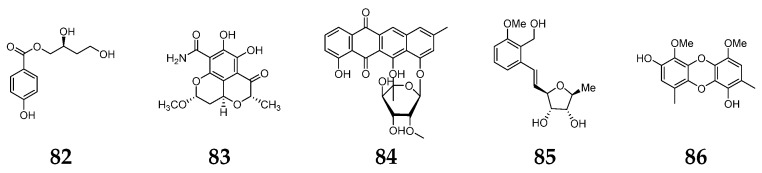
Chemical structures of compounds **82**–**86**.

One new aromatic compound, (*S*)-2,4-dihydroxy-1-butyl-(4-hydroxy) benzoate (**82**) (Chart 15), was characterized from *Penicillium auratiogriseum* associated with the marine sponge *Mycale plumose* (Qingdao, China). This metabolite was shown to exhibit potent cytotoxic effect on tsFT210 cells with an MIC (minimum inhibitory concentration) value of 8.0 µg/mL [46]. Fractionation of the extract of a fermentation broth of a marine sponge-derived strain of *Streptomyces* sp., SpD081030ME-02 (collected at offshore of Ishigaki City, Japan) afforded a new compound JBIR-58 (**83**) (Chart 15), exhibited cytotoxic effect on HeLa cells with an IC_50_ value of 28 µM [47]. A new anthracycline, tetracenoquinocin (**84**) (Chart 15), was metabolized by the *Streptomyces* sp. Sp080513GE-26 associated with *Haliclona* sp. (Tateyama City, Japan) and showed weaker cytotoxicity against human cervical carcinoma HeLa cells and acute myelogenous leukemia LH-60 cells with IC_50_ values of 120 and 210 µM, respectively [48]. From a *Porifera* sponge-derived strain, *Emericella variecolor*, one new aromatic varitriol (**85**) (Chart 15) was identified and found to have a broad spectrum of anti-proliferative effects [49]. The new dibenzo(1,4)dioxin **86** (Chart 15) was isolated from *Aspergillus versicolor* Hmp-F48, associated with marine sponge *Hymeniacidon perleve,* and exhibited moderate inhibitory activity against HL-60 cells with IC_50_ 3.62 µM [45].

## 6. Lactones

**Chart 16:**
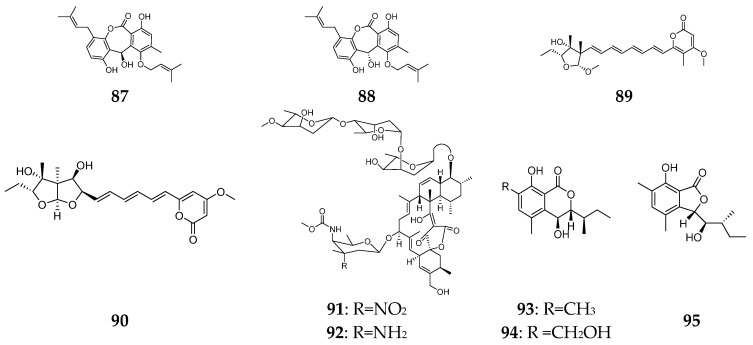
Chemical structures of compounds **87**–**95**.

Chemical analysis of *Emericella variecolor* XSA-07-2 isolated from the South China Sea sponge *Cinachyrella* sp. afforded four new lactones varioxiranols I–L (**87**–**90**) (Chart 16) with different scaffolds. Cytotoxic tests suggested that these isolates showed moderate cytotoxic activities against human colon carcinoma (HCT-116), liver hepatocellular carcinoma (HepG2), gastric cancer (BGC-823), lung cancer stem cells (NCI-H1650), and human ovarian cancer (A2780) [50]. One bacterial strain, *Streptomyces carnosus,* obtained from marine sponges *Hymeniacidon* sp. (collected from coastal waters of East China) was found to produce two new lactones: lobophorin C (**91**) and D (**92**) (Chart 16). Compound **91** displayed potent cytotoxic activity against the cell proliferation of hepatoma 7402 with an IC_50_ value of 0.6 µg/mL. In addition, **92** had a strong inhibitory effect on the growth of the human breast cancer cell line MDA-MB 435 with IC_50_ 7.5 µM [51]. Three new benzolactones, chrysoarticulins A–C (**93**–**95**) (Chart 16), were isolated from the culture broth of *Chrysosporium articulatum* collected from an unidentified dictyoceratid sponge (Gagu-do, Korea). All these compounds exhibited weak cytotoxicity against tumor cell lines K562 and A549 [52].

## 7. Steroids

**Chart 17:**
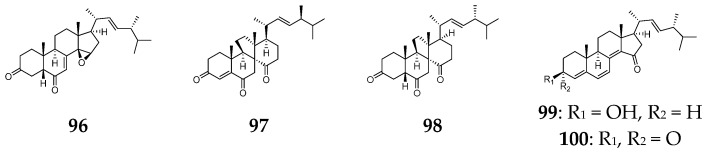
Chemical structures of compounds **96**–**100**.

Five structurally unusual steroids, **96**, **97**, **98**, **99**, and **100** (Chart 17), were metabolized by one marine strain of *Gymnasella dankaliensis* isolated from the sponge *Halichondria japonica* (Osaka Bay, Japan) and exhibited significant and marginal growth inhibition against the lymphocytic leukemia P388 cell line with ED_50_ values of 1.6, 2.2, 2.8, 0.9, and 2.5 µg/mL, respectively [29,53,54].

## 8. Miscellaneous Compounds

**Chart 18:**
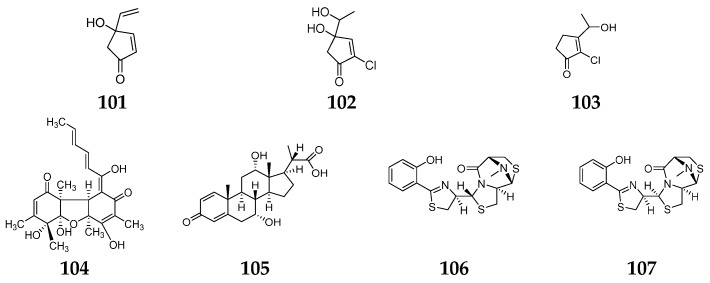
Chemical structures of compounds **101**–**107**.

Novel metabolites trichodenones A–C (**101**–**103**) (Chart 18) were detected in the culture broth of *Trichoderma harzianum* OUPS-N 115 originally separated from marine sponge *Halichondria okadai* (collected in the Tanabe Bay, Japan) and shown to possess strong cytotoxicity against P388 cells [55]. Chemical investigation of the fungal strain *Penicillium citrinum* SpI080624G1f01, derived from the *Demospongiae* sponge (collected from Ishigaki Island, Japan), afforded a new compound, JBIR-59 (**104**) (Chart 18), which had l-glutamate toxicity against tumor cell line N18-RE-105 with an EC_50_ value of 71 µM [56]. One novel sterol bendigole **105** (Chart 18) produced by *Actinomadura* sp. SBMs009 from the marine sponge *Suberites japonicus* displayed a moderate cytotoxic effect on the L929 cells with an IC_50_ value of 30 µM [57]. Two new structurally unique compounds bearing a nitrogen and sulfur-containing tricyclic ring system, ulbactin F (**106**) and its diastereomeric isomer ulbactin G (**107**) (Chart 18), were isolated from the culture extract of *Brevibacillus* sp. associated with an unidentified marine sponge (Iwate, Japan). Bioassay testing indicated that **106** and **107** had a strong inhibitory effect on epidermoid carcinoma cell line A431 at non-cytotoxic concentrations with IC_50_ values of 6.4 and 6.1 µM, respectively [58].

In summary, microorganisms associated with marine sponges are a prolific source of novel cytotoxic natural products with rich chemical structures. The utilization of natural products as sources of new drugs is still alive and well, especially in the area of cancer [59]. Generally, any cytotoxic chemical with an IC_50_ or ED_50_ value <1 µM has great potential for application in the discovery of new anti-tumor drugs/leads, for example, tetracyclic alkaloid **31** and dankastatin C (**57**). These candidates may play an important role in defeating human cancer.
